# Supplementary material for: Mycobacterium tuberculosis-dependent Monocyte Expression Quantitative Trait Loci and Tuberculosis Pathogenesis
Source: medRxiv. 2023 Aug 29:2023.08.28.23294698. Preprint. [Version 1] doi: 10.1101/2023.08.28.23294698 (PMC10491362; doi:10.1101/2023.08.28.23294698)
Supplement: Supplement 7 [file media-7.docx]

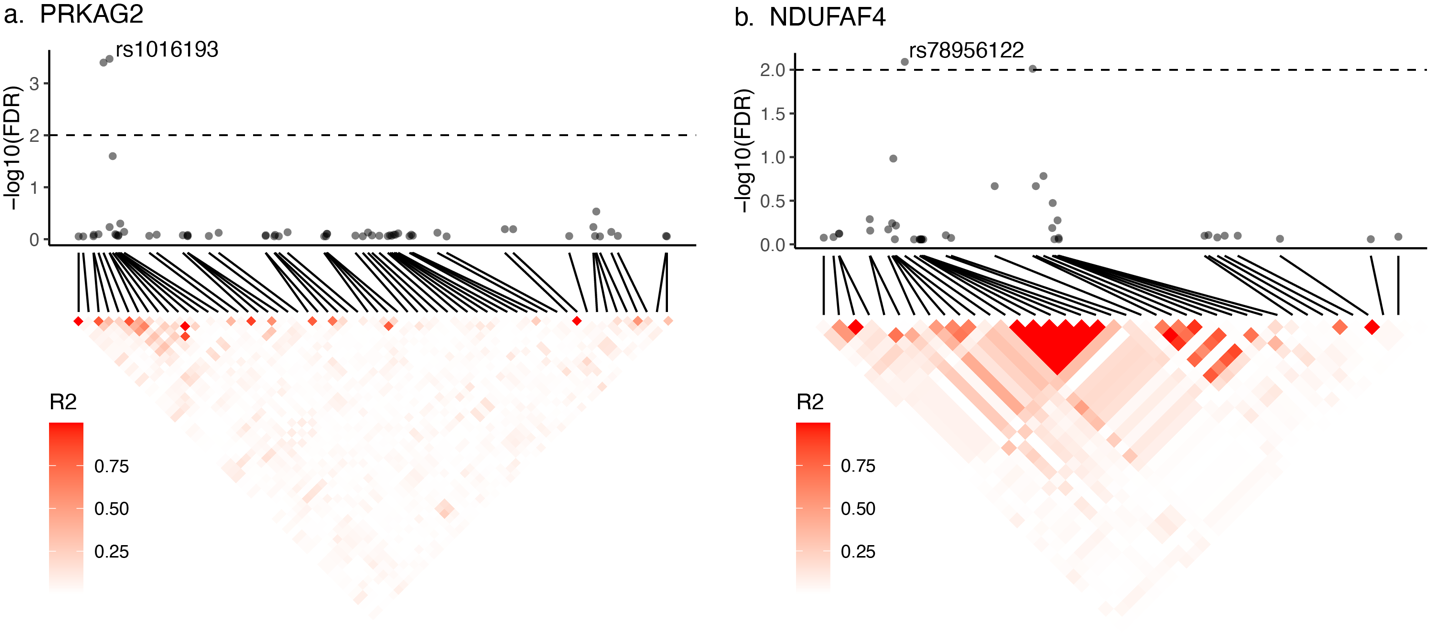


**Figure S2. Pattern of linkage disequilibrium.** PRKAG2 and NDUFAF4 had 2 cis eQTLs in high linkage disequilibrium (LD). (top) eQTL significance for SNPs within 1 MB of (a) PRKAG2 and (b) NDUFAF4. X-axis indicates chromosome position of the eQTLs. Horizontal dashed line indicates FDR = 0.01 and the lead SNP is labeled. (bottom) Heatmap indicating R^2^ LD for SNPs in this region.
